# Altered low-frequency brain rhythms precede changes in gamma power during tauopathy

**DOI:** 10.1016/j.isci.2022.105232

**Published:** 2022-09-28

**Authors:** Fabio R. Rodrigues, Amalia Papanikolaou, Joanna Holeniewska, Keith G. Phillips, Aman B. Saleem, Samuel G. Solomon

**Affiliations:** 1 UCL Institute of Behavioural Neuroscience, Department of Experimental Psychology, University College London, London WC1H 0AP, UK; 2 Eli Lilly, Research and Development, Erl Wood, Surrey GU20 6PH, UK

**Keywords:** Biological sciences, Neuroscience, Clinical neuroscience, Cognitive neuroscience

## Abstract

Neurodegenerative disorders are associated with widespread disruption to brain activity and brain rhythms. Some disorders are linked to dysfunction of the membrane-associated protein Tau. Here, we ask how brain rhythms are affected in rTg4510 mouse model of tauopathy, at an early stage of tauopathy (5 months), and at a more advanced stage (8 months). We measured brain rhythms in primary visual cortex in presence or absence of visual stimulation, while monitoring pupil diameter and locomotion to establish behavioral state. At 5 months, we found increased low-frequency rhythms during resting state in tauopathic animals, associated with periods of abnormally increased neural synchronization. At 8 months, this increase in low-frequency rhythms was accompanied by a reduction of power in the gamma range. Our results therefore show that slower rhythms are impaired earlier than gamma rhythms in this model of tauopathy, and suggest that electrophysiological measurements can track the progression of tauopathic neurodegeneration.

## Highlights

• rTg4510 animals have increased low-frequency power in V1 at early stages of disease
• At later stages, following degeneration, V1 gamma power is reduced
• Low-frequency rhythms are more strongly coupled with each other in tauopathy

## Graphical abstract

**Figure:**
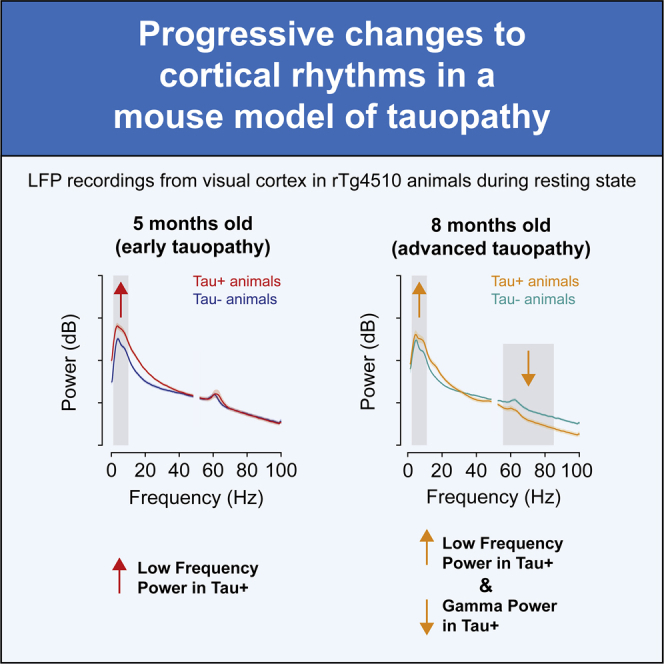

## Introduction

Several neurodegenerative disorders, including Alzheimer's disease (AD), are associated with malfunction of the microtubule-associated protein Tau. In tauopathy, Tau is hyperphosphorylated, disrupting neuronal function (Crimins et al., 2011; Kopeikina et al., 2013; Roy et al., 2005) and impairing the stability of synapses (Hoover et al., 2010; Jackson et al., 2017). These impairments to neuronal structure and function should have correlates in the electrical activity of the brain, so the electroencephalogram (EEG) and related measurements could offer potentially powerful, low-cost tools for detecting and tracking the functional impact of tauopathy.

Neuronal activity often coheres into brief rhythms (Buzsaki and Draguhn, 2004). These rhythms indicate synchronization of neural activity, which is likely to be important for brain function. Synchronization at higher frequencies is hypothesized to be important for cognitive processing (e.g. Bastos et al., 2015), while synchronization at lower frequencies is hypothesized to be a default state of cortical networks (Sanchez-Vives et al., 2017), and is likely to be important for functions including homeostasis and plasticity (e.g. Timofeev et al., 2020; Vyazovskiy et al., 2011). EEG measurements from patients with AD indicate increased power at low frequencies (delta, ca. 2–4 Hz) (Coben et al., 1983; Huang et al., 2000), and reduced power at higher frequencies including alpha, beta, and gamma-bands (for a review see Babiloni et al., 2020). However, the contribution of tauopathy to these changes is unclear, and our understanding of the temporal progression of these disruptions is limited.

In preclinical mouse models of tauopathy, low-frequency EEG oscillations are increased (Das et al., 2018), fMRI resting state networks are disrupted (Green et al., 2019), and coupling between hippocampal and prefrontal cortical local field potentials (LFPs) is disrupted (Ahnaou et al., 2017). In the well-characterized rTg4510 model of tauopathy, hippocampal LFP (Ciupek et al., 2015) and cortical EEG are disrupted (Holton et al., 2020), and strong low-frequency oscillations emerge in LFP recordings from frontal cortex (Menkes-Caspi et al., 2015) even early in tauopathy. Only limited work, at more advanced stages of tauopathy, has characterized impact on gamma-band oscillations (30–100 Hz) and found reduced gamma in the hippocampus (Booth et al., 2016b) and entorhinal cortex (Booth et al., 2016a) of rTg4510 mice. Consequently, it is not clear if changes in oscillations at low and high frequencies occur together in tauopathy, or if one precedes the other. This is important, because disruptions that arise earlier in tauopathy may provide better diagnostic or therapeutic targets.

Frontal cortex and hippocampal areas are associated with complex cognitive functions that make it difficult to make finely controlled measurements of function in awake animals. Sensory brain areas, such as visual cortex, offer the opportunity to characterize the influence of tauopathy on low- and high-frequency brain oscillations in head-fixed animals, during presence and absence of external stimulation (visual stimulus), while closely monitoring behavioral states such as arousal and locomotion (Niell and Stryker, 2010; Reimer et al., 2014; Vinck et al., 2015). EEG and LFP signals from the mouse primary visual cortex (V1) are dominated by low-frequency oscillations (2–6 Hz) (Senzai et al., 2019) with additional oscillations in the gamma range (Buzsaki and Draguhn, 2004), including components driven by thalamic inputs (McAfee et al., 2018; Saleem et al., 2017; Storchi et al., 2017).

In this study, we evaluated the influence of tauopathy on LFP signals in visual cortex by analyzing recordings from V1 in the rTg4510 mouse model. Recordings were made at 5 months, a relatively early stage before pronounced pathology, or at 8 months, when tauopathy is more advanced (Ramsden et al., 2005; Santacruz et al., 2005). We found a pronounced increase in low-frequency oscillations early in tauopathy, which was linked to the occurrence of abnormal periods of high cortical synchronization. Gamma power and stimulus-induced gamma oscillations were reduced only at more advanced stages of neurodegeneration. We conclude that changes in low-frequency spectral power precede changes in gamma power in the rTg4510 model of tauopathy.

## Results

**Figure 1:**
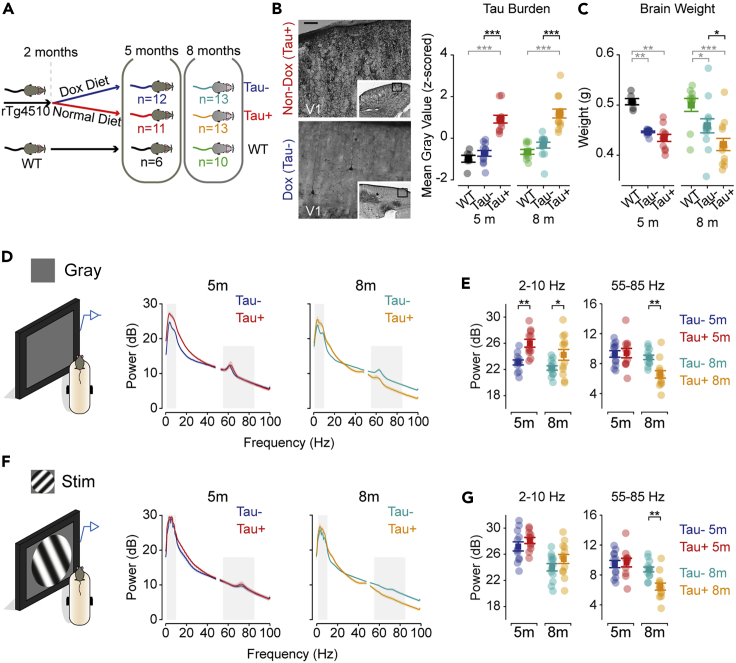
Visual cortical oscillations are disrupted in the rTg4510 mouse model (A) rTg4510 mice were maintained on a regular diet (Tau+, 24 animals) or switched to a doxycycline-containing diet from 2 months to arrest mutant *tau* expression (Tau-, 25 animals). (B) *Left*: AT8-staining for phosphorylated Tau in representative 5-month-old Tau+ and Tau- animals. Scale bar 100 μm. *Right*: Tau burden (mean image intensity) was higher in Tau + mice than Tau- and WT (5m, p= 8 × 10^−9^; 8m: p = 1 × 10^−8^). (C) Brain weight of Tau+ was less than Tau- at 8 months (p = 0.030) but not 5m (p = 1.00). Brain weight of both Tau- (5m, p = 0.004; 8m, p = 0.023) and Tau+ (5m, p = 0.001; 8m, p = 7 × 10^−6^) was less than WT. See also Figure S1 for estimates of visual cortical atrophy. (D) Power spectrum of LFP recordings obtained from visual cortex in head-fixed mice during presentation of gray screen. Gray rectangles highlight the average power within low (2–10 Hz) and gamma (55–85 Hz) frequency ranges. (E) Average power during presentation of gray screen, at low frequencies (left) and gamma frequencies (right). Low frequency power in Tau- animals was less than in Tau+ animals at 5 (*F*_(2,59)_ = 11.204; p = 0.001) and 8 months (p = 0.022); gamma frequency power was less in Tau+ than Tau- animals at 8 months (*F*_*(*2,59)_ = 7.707; p = 0.003) but not 5 months (p = 1.00). (F) Same as D, but during presentation of a sinusoidal grating that reversed contrast at 2 Hz. (G) Same as E, but for average power during stimulus presentation. Low-frequency power was similar in Tau- and Tau+ animals at 5 and 8 months (*F*_*(*2,59)_ = 1.653; p = 0.200); gamma frequency power was less in Tau+ than Tau- animals at 8 months (*F*_(2,59)_ = 7.156; p = 0.002) but not 5 months (p = 1.00). Panels (A–C) adapted from Papanikolaou et al. (2022). Data in (B, C, E, and G) are represented as mean ±1 s.e.m using opaque symbols, and transparent symbols show individual animals. Shaded area in D and F represents ±1 s.e.m (usually too small to be seen) from the mean (line). All comparisons were performed using a two-way ANOVA. Statistical comparisons in black highlight comparisons between Tau- and Tau+ animals, while statistical comparisons in gray are for comparisons of any Tau group and WT littermates. ∗p < 0.05; ∗∗p < 0.01; ∗∗∗p < 0.001.

We measured the LFP from electrodes chronically implanted into layer 4 of V1 of awake rTg4510 animals and their wild-type (WT) littermates. We have reported analyses of visual evoked potentials, obtained from the same recordings, elsewhere (Papanikolaou et al., 2022). At 2 months of age, half the rTg4510 animals were transferred to a doxycycline-containing diet to suppress the expression of the transgene (henceforth Tau- animals), while the remaining animals were maintained on a normal diet (Tau+) (Figure 1A). As expected, phosphorylated tau burden (as indicated by staining for AT8 antibody) was higher in 5 months and 8 months Tau+ animals than in Tau- or WT animals (Figure 1B) (Ramsden et al., 2005; Santacruz et al., 2005). The brain weight of Tau+ and Tau- animals was similar at 5 months, and both were smaller than those of WT littermates, but at 8 months the brain weight of Tau+ animals was smaller than that of Tau- animals, indicating degeneration in Tau+ animals between 5 and 8 months (Figure 1C). The doxycycline treatment was therefore effective in suppressing expression of the mutant tau in Tau- animals, as expected from previous work (Blackmore et al., 2017). To establish that this atrophy was also present within V1, we measured cortical thickness in histological brain sections (Figure S1). At 5 months, cortical thickness of V1 was similar in all phenotypes (*F*_(5,60)_ = 6.793, p = 1.00). At 8 months, V1 was thinner in Tau+ than in either Tau- (p = 0.002) or WT (p = 3.3 × 10^−5^).

### Visual cortical oscillations are disrupted in rTg4510 model of tauopathy

We measured LFP from head-fixed animals over seven consecutive days, while the animals passively viewed either a gray screen or a patterned visual stimulus. On each day, the animal was initially presented with a uniform gray screen, after which we interleaved presentations of a large, flickering grating and additional presentations of the gray screen. We subjected the LFP recording on each day to multitaper spectral analysis (see STAR Methods), which provided estimates of the power spectrum of the LFP at a resolution of 1s. We then collapsed data across epochs of gray screen and stimulus presentation, and averaged observations across days, to produce two estimates of the power spectrum in each animal.

The power spectrum of visual cortical LFP was altered in both early and more advanced stages of tauopathy. We first analyzed LFP signals during the presentation of a gray screen. We found a pronounced increase in power at low- (2–10 Hz) and mid-range (10–30 Hz) frequencies in 5- and 8-month-old Tau+, compared to Tau- (Figure 1D) and WT animals (not shown). For example, low-frequency power was 23.1 ± 0.4 dB (mean, 1 s.e.m) and 26.0 ± 0.6 dB in 5-month-old Tau- and Tau+ animals, respectively (*F*_(2,59)_ = 11.204; p = 0.001; Figure 1E); at 8 months of age, low-frequency power was instead 22.2 ± 0.3 and 24.2 ± 0.8 dB (p = 0.022). At 8 months, increased power at these lower frequencies in Tau+ was accompanied by a decrease in power at gamma frequencies (5 months: Tau- was 9.3 ± 0.4 dB and Tau+ 9.4 ± 0.6; *F*_*(*2,59)_ = 7.707, p = 1.00; 8 months: 8.8 ± 0.3 vs. 6.6 ± 0.5; p = 0.003). During periods of visual stimulus presentation (Figures 1F and 1G), power at lower frequencies was similar between Tau+, Tau-, and WT (not shown) animals, at both 5 and 8 months. Because the contrast of the stimulus reversed at a rate of 2 Hz, it elicited strong visually evoked potentials in all animals (see Papanikolaou et al., 2022), and it is therefore difficult to distinguish the contribution of stimulus-induced power changes, from those associated with ongoing cortical activity. Visual stimulation at 8 months did not, however, restore high-frequency activity in Tau+ animals, suggesting that the power reduction at high frequencies cannot be rescued by slow visual stimulation.

### Impact of tauopathy on visual cortical activity is more prominent during resting state

**Figure 2:**
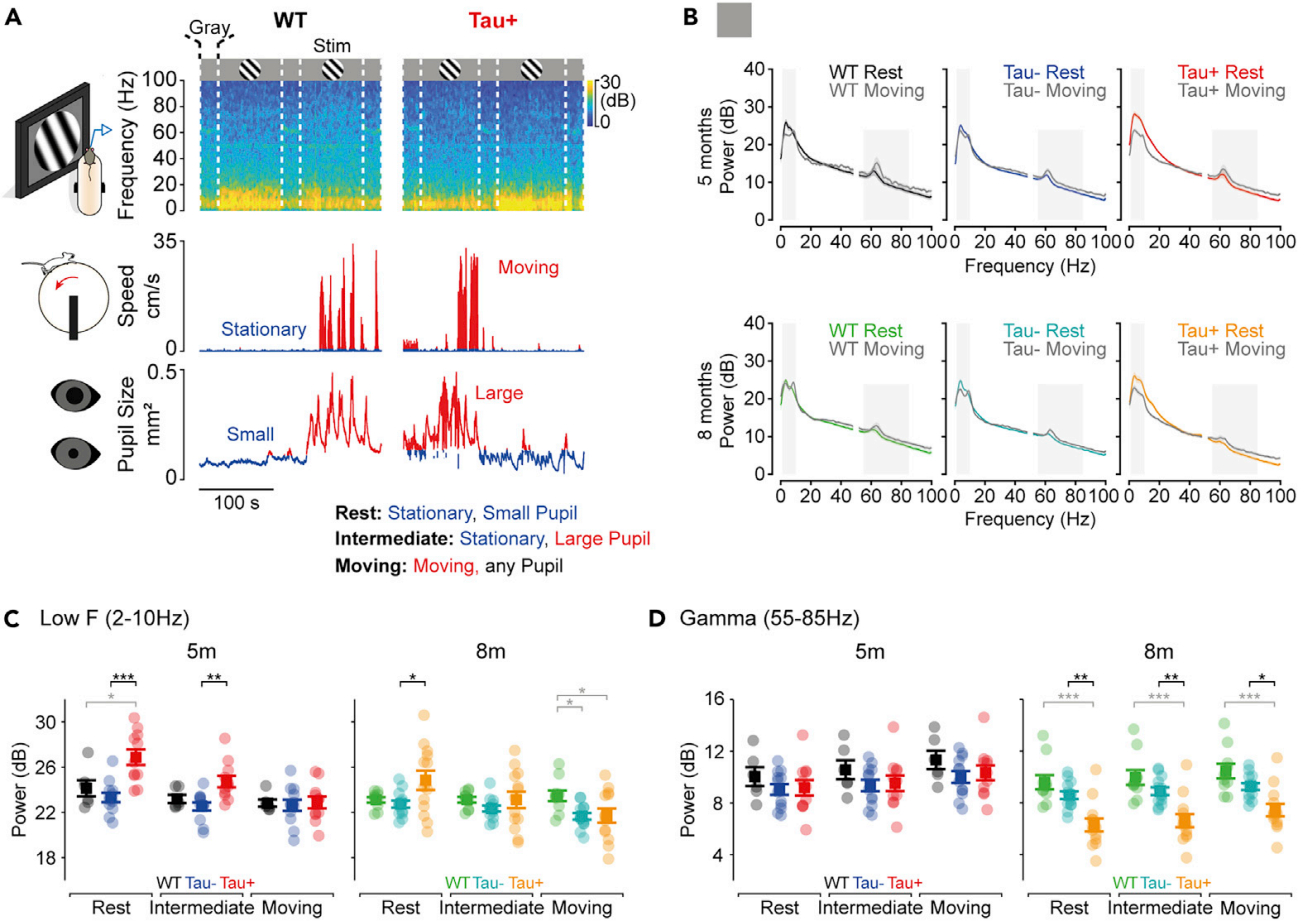
Greater impact of tauopathy on low-frequency rhythms during rest (A) Spectrograms of the LFP were divided into “rest”, “intermediate”, and “moving” epochs defined by simultaneous measurement of locomotion speed and pupil diameter. (B) Power spectra obtained by averaging within and then across animals in each group, for rest and moving epochs. Tau+ animals show increased power at low frequencies during rest. Gray rectangles highlight the average power within low (2–10 Hz) and gamma (55–85 Hz) frequency ranges. (C) Average power at low frequencies (2–10 Hz) during gray screen intervals. Resting state power was greater in Tau+ than controls at 5 and 8 months (*F*_(2.432,71.747)_ = 15.347; WT x Tau+ 5m, p = 0.022; Tau-x Tau+ 5m, p = 1.54 × 10^−4^; WT x Tau+ 8m, p = 0.114; Tau-x Tau+ 8m, p = 0.023). Intermediate state power was greater in Tau+ than Tau- mice at 5 months (p = 0.005). All other intergroup comparisons were p > 0.05. (D) Average power at gamma frequencies (55–85 Hz). Power in 8m Tau+ animals was less than in controls during rest (*F*_(2.855,84.226)_ = 5.757; WT x Tau+ 8m, p = 3 × 10^−5^; Tau-x Tau+ 8m, p = 0.002), intermediate (WT x Tau+ 8m, p = 4 × 10^−5^; Tau-x Tau+ 8m, p = 0.003), and moving states (WT x Tau+ 8m, p = 1 × 10^−4^; Tau-x Tau+ 8m, p = 0.017). Shaded area in B represents ±1 s.e.m (usually too small to be seen) from the mean (line). Data in C and D are represented as mean ±1 s.e.m using opaque symbols, and transparent symbols show individual animals. All comparisons were performed using a Mixed ANOVA. Statistical comparisons in black highlight comparisons between Tau- and Tau+ animals, while statistical comparisons in gray are for comparisons of any Tau group and WT littermates. ∗p < 0.05; ∗∗p < 0.01; ∗∗∗p < 0.001.

Measurements of cortical electrical activity in patients with AD show larger disruptions during periods of quiet rest (Babiloni et al., 2009; de Haan et al., 2008). We therefore asked if the changes in spectral power that we observed in the Tau+ animals were also more prominent during rest. During our measurements, animals were able to move freely over a styrofoam wheel. We recorded the animals’ movement and pupil diameter to monitor the behavioral state of the animal. From these measurements, we defined resting, intermediate, and moving states (Figure 2A): “resting” state was when the animal was stationary and pupil size was smaller than the mean size over that recording session; “intermediate” state was when the animal was stationary and pupil size was larger than the mean size over that recording session; and “moving” state was when the animal was moving, irrespective of pupil size. We generated power spectra across moving, intermediate, and rest periods each day, in the presence and absence of visual stimulus.

The increase in low frequency oscillations in Tau+ animals was more pronounced during rest (Figures 2B and 2C). At rest, the spectral power at low frequencies (averaged over 2–10 Hz) was 13% larger in Tau+ than in control (Tau- and WT) animals at 5 months, and 8% larger at 8 months. During movement, low-frequency power was reduced in all animal groups, and there were no differences between Tau+ and control animals at either 5 or 8 months (Figure 2C). In intermediate states, we found reduced low-frequency power compared to rest periods, which suggests that changes in low-frequency power during movement can be related to arousal: at 5 months, low-frequency power was reduced during intermediate states by 8% in Tau+, 4% in Tau-, and 3% in WT; at 8 months, these were 7% in Tau+, 2% in Tau-, and 0% in WT [comparative statistics can be found in Table S1]. The result was that (during intermediate state) low-frequency power was similar in all animal groups at 8 months, and was slightly larger in Tau+ than Tau- (but not WT) at 5 months. Thus, arousal is sufficient to abolish most of the differences in low-frequency power between Tau+ and control animals. We showed that presentation of flickering visual stimuli abolished differences in low-frequency power between Tau+ and Tau- animals (Figure 1)—this was also the case when we considered moving, intermediate, and resting periods separately (not shown).

### More advanced tauopathy disrupts high gamma-band activity

**Figure 3:**
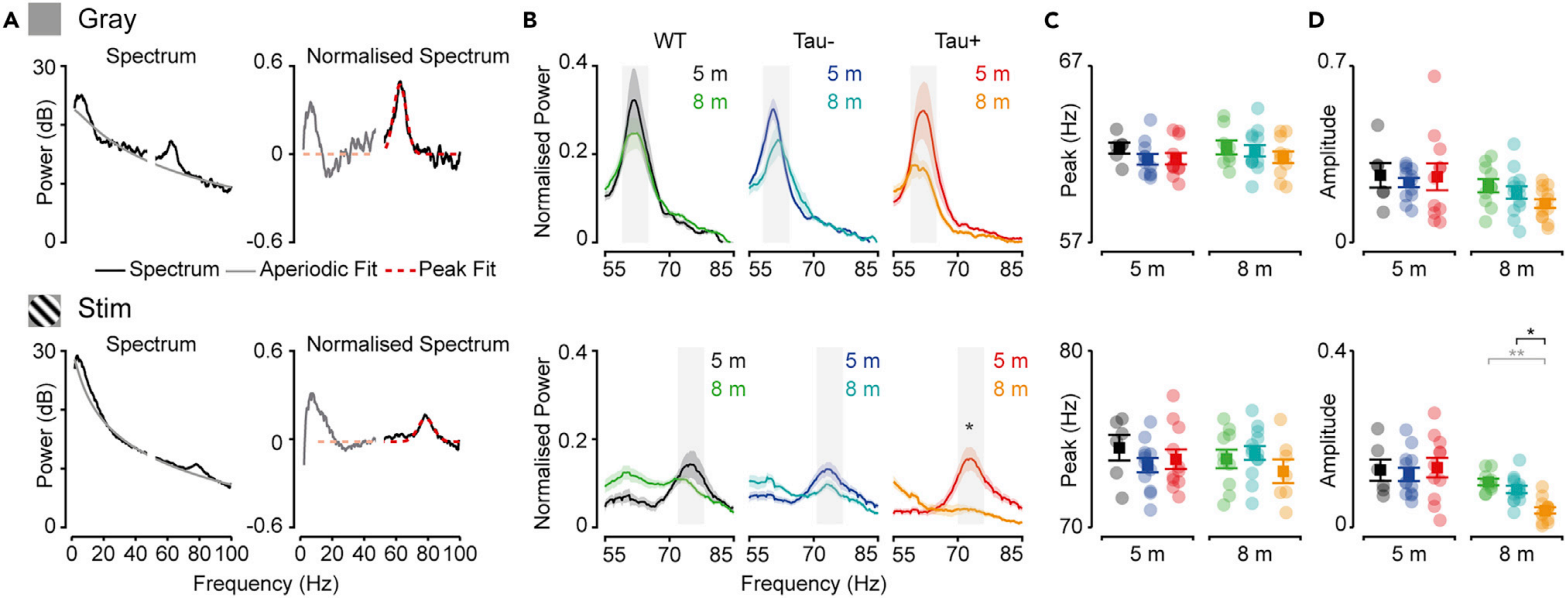
Impaired gamma oscillations in more advanced stages of tauopathy (A) Normalized power spectra (right panel) were produced by subtracting the aperiodic component (left panel, gray line) from the power spectrum. (B) Average normalized spectrum during rest, focusing on the frequency ranges in which gamma peaks were observed during presentation of gray screen (top) and stimulus (bottom). Amplitude of stimulus-induced gamma was smaller in 8-month Tau+ animals (*F*_(2,59)_ = 3.822; p = 3 × 10^−6^). Gray rectangles indicate approximate 6 Hz windows used to calculate mean normalized power in D. (C) There was no difference in the center frequency of detected gamma-rhythm, during presentation of gray screen or stimulus. (D) Mean normalized power for a 6 Hz range around the mean center frequencies for the relevant gamma peaks. Tau+ mice showed smaller mean power for stimulus-induced gamma at 8 months (WT x Tau+, p = 0.004; Tau-x Tau+, p = 0.032), but not at 5 months, or during gray screen periods (*F*_(2,59)_ = 0.870, p = 0.589). Shaded area in B represents ±1 s.e.m from the mean (line). Data in C and D are represented as mean ±1 s.e.m using opaque symbols, and transparent symbols show individual animals. All comparisons were performed using a two-way ANOVA. Statistical comparisons in black highlight comparisons between Tau- and Tau + animals, while statistical comparisons in gray are for comparisons of any Tau group and WT littermates. ∗p < 0.05; ∗∗p < 0.01.

Gamma-band activity in the visual cortex can be decomposed into multiple sub-bands (Chen et al., 2017; Engel et al., 2001; Veit et al., 2017). Activity at some frequencies (e.g., “narrowband gamma”; ca. 55–65 Hz; (Saleem et al., 2017)) may arise by entrainment of visual cortex to oscillations that are already present in the thalamocortical input (McAfee et al., 2018; Saleem et al., 2017; Storchi et al., 2017). Activity in other sub-bands may be more reliant on cortico-cortical signals, or reflect the activity of local interneurons (e.g., parvalbumin interneuron-driven oscillations at ca. 70–80 Hz) (Veit et al., 2017). We showed that overall gamma-band activity (30–100 Hz) is reduced in 8-month Tau+ animals, during presentation of both gray screen and visual stimulus (Figure 1). However, the power spectrum in the high gamma-band range also showed clear peaks, and their center frequencies were different in the gray screen and stimulus conditions (Figure 3A). As expected from previous work (Saleem et al., 2017), gamma power peaked in a narrow range around 55–65 Hz during the gray screen condition. During the presentation of the visual stimulus, this peak disappeared and another emerged at higher frequencies, around 70–80 Hz (McAfee et al., 2018; Veit et al., 2017). To characterize these peaks, we first calculated the aperiodic component of the power spectra, using standard methods (Donoghue et al., 2020) (Figure 3A). The aperiodic component decreases monotonically as a function of frequency, and we subtracted (in dB) this aperiodic component from the raw spectrum to produce a normalized spectrum (Figure 3B).

Evidence for the presence of each of the two gamma peaks could be found in individual animals from all groups, but the visual-stimulus-induced gamma peaks appeared more susceptible to tauopathy than those occurring during gray screen. We used normalized spectra to characterize the changes in gamma peaks. We extracted the center frequency of the best-fitting Gaussian for each condition in each animal. We found that the center frequencies during gray screen, or during stimulus presentation, were similar across animal groups (Figure 3C). To compare the amplitude of activity around these peaks, we calculated the mean amplitude of the normalized power spectra in a 6 Hz range centered on the mean peak frequency for each animal group. The amplitude of gamma peaks during gray screen was similar across age and groups (*F*_(2,59)_ = 0.870, p = 0.424). By contrast, the amplitude of stimulus-induced gamma reduced from 5 to 8 months in all groups, particularly in Tau+ animals, such that the mean amplitude was smaller in 8-month Tau+ than in 8-month Tau- *(F*_(2,59)_ = 3.822; p = 0.032) or WT (p = 0.004) animals. We conclude that, under these conditions, stimulus-induced gamma peaks are more susceptible to tauopathy than the narrowband gamma oscillations that occur in the absence of a visual stimulus.

### Visual cortex is more synchronized during tauopathy

**Figure 4:**
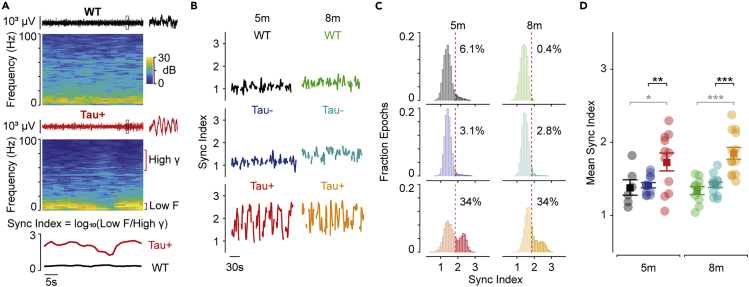
Increased synchronization of visual cortical activity during tauopathy (A) *Upper panels*: example LFP waveforms and associated spectrograms for WT and Tau+ animals during presentation of a gray screen. Inset expands the 1s period highlighted in the main LFP trace. *Lower panel*: synchronization (‘sync’) index is the logarithm of the ratio between power at low (2–10 Hz) and high (55–85 Hz) frequencies. Larger sync indices represent increased low frequency power. (B) Sync index for concatenated epochs of resting state. Tau+ animals show intermittent epochs of high synchronization at 5 and 8 months. (C) Distributions of sync index over all rest periods in all animals. The 95^th^ percentile of the sync index for WT pooled over age (red dashed line) was 1.85. The sync index more frequently exceeded this threshold in Tau+ than Tau- animals. (D) Mean sync index during rest in each animal. Tau+ animals showed higher sync indices at both ages (*F*_(4.372,128.981)_ = 6.800; WT 5m x Tau+ 5m, p = 0.024; Tau- 5m x Tau+ 5m, p = 0.009; WT 8m x Tau+ 8m, p = 3.6 × 10^−5^; Tau- 8m x Tau+ 8m, p = 1.7 × 10^−4^). Data in D are represented as mean ±1 s.e.m using opaque symbols, and transparent symbols show individual animals. All comparisons were performed using a two-way ANOVA. Statistical comparisons in black highlight comparisons between Tau- and Tau+ animals, while statistical comparisons in gray are for comparisons of any Tau group and WT littermates. ∗p < 0.05; ∗∗p < 0.01; ∗∗∗p < 0.001.

We showed that low-frequency power is increased in Tau+ animals during rest. Inspection of individual spectrograms further showed that the resting state in Tau+ animals (Figure 4A) was organized into epochs during which there was an increase in power across a wide band of low frequencies. The LFP traces in these epochs were characterized by strong and coherent low-frequency oscillations (Figure 4A). Spectrograms obtained from Tau- and WT animals showed a more variegated structure of power at low frequencies (Figure 4A) and lacked the clear transitions into and out of these low-frequency states. To characterize the fluctuations in low-frequency power, we calculated a synchronization (“sync”) index as the base 10 logarithm of the ratio of low frequency (2–10 Hz) to high gamma (55–85 Hz) power in each time bin. We note that by synchronization, we mean cortical activity dominated by low-frequency rhythms, like those that occur during sleep, or quiet wakefulness (Harris and Thiele, 2011). Therefore, higher sync-indices indicate relatively stronger power in the low-frequency range.

We found high and variable sync-index in Tau+ animals, with epochs of strong synchronization interspersed by epochs of weak synchronization (Figure 4B); the strongly synchronized states were more prominent in some Tau+ animals than in others. The sync-index was smaller and less variable in Tau- and WT animals. To illustrate this, we estimated a threshold sync-index (which was 1.85) as the mean +2 s.d. of the distribution obtained from pooled WT animals. The fraction of epochs exceeding this threshold was approximately 3% in Tau- animals, and 34%–35% in Tau+ animals (Figure 4C). Consequently, the sync-index was on average higher in Tau+ animals than Tau- (Figure 4D; *F*_(4.372,128.981)_ = 6.800; 5 months, p = 0.009; 8 months, p = 1.7 × 10^−4^), or WT (5 months, p = 0.024; 8 months, p = 3.6 × 10^−5^).

**Figure 5:**
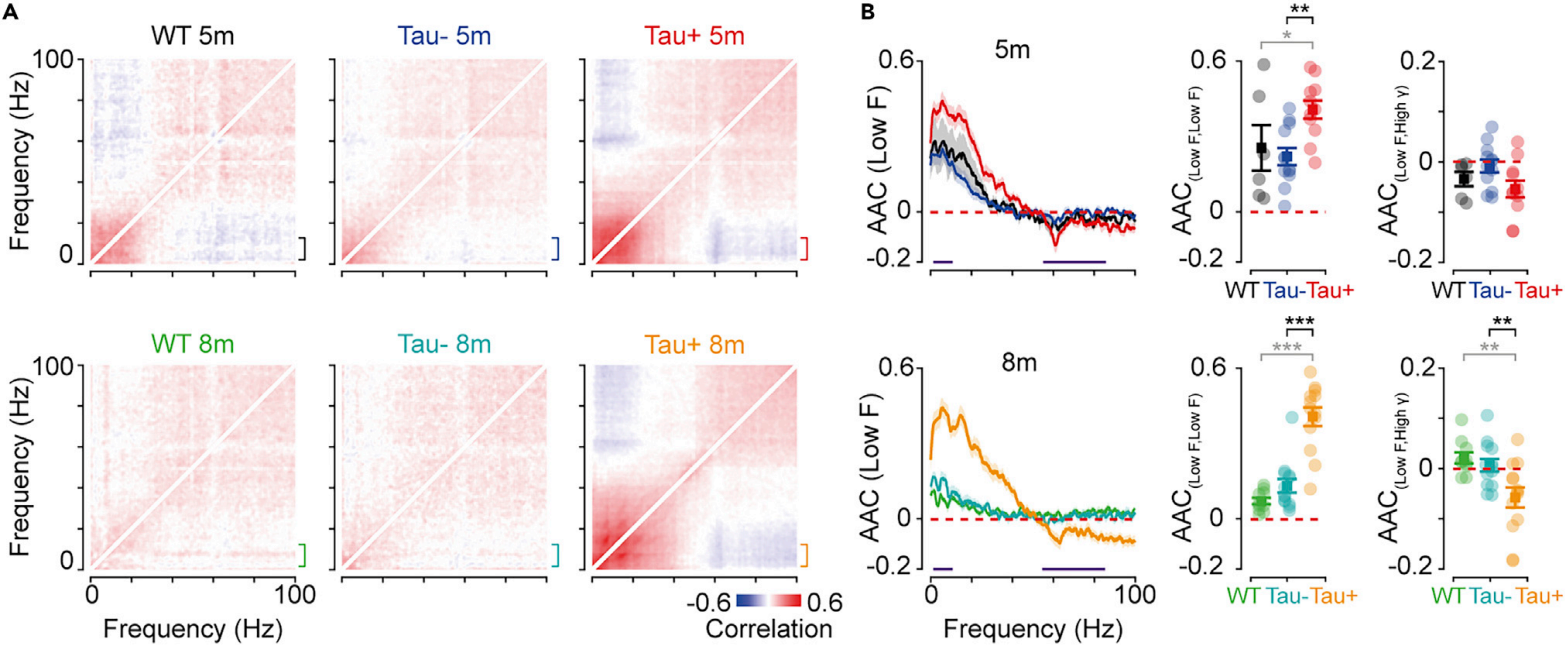
Covariation of low frequency activity in Tau + animals (A) Average amplitude-amplitude correlation (AAC) between different frequencies, during rest periods. Brackets indicate a band of low frequencies (2–10 Hz). (B) *Left*: Average AAC for a band of low frequencies (2–10 Hz; indicated by the brackets in A), against all other frequencies. *Middle*: Correlations between low frequencies were stronger in Tau+ animals at both 5 and 8 months (*F*_(2,59)_ = 27.827; WT x Tau+ 5m, p = 0.052; Tau-x Tau+ 5m, p = 0.002; WT x Tau+ 8m, p = 5 × 10^−8^; Tau-x Tau+ 8m, p = 1 × 10^−6^). *Right*: Anti-correlations between low frequencies and high frequencies (55–85 Hz) were stronger in Tau + animals at 8 months (*F*_(2,59)_ = 7.915; WT x Tau+ 8m, p = 0.002; Tau- x Tau+ 8m, p = 0.007). Data in B are represented as mean ±1 s.e.m using opaque symbols, and transparent symbols show individual animals. All comparisons were performed using a two-way ANOVA. Statistical comparisons in black highlight comparisons between Tau- and Tau+ animals, while statistical comparisons in gray are for comparisons of any Tau group and WT littermates. ∗p < 0.05; ∗∗p < 0.01; ∗∗∗p < 0.001.

Fluctuations in the sync-index may be driven by fluctuations in low-frequency power, gamma power, or both. We therefore analyzed the temporal correlation in power at different frequencies (amplitude-amplitude coupling, or AAC (Bruns et al., 2000; Siegel et al., 2012)). For each session, we calculated the temporal correlation between all combinations of frequencies (while the animal was at rest), then averaged across sessions and animals (Figure 5A). Similar frequencies were generally positively coupled—that is, power at similar frequencies modulated in synchrony. Power at very different frequencies was instead usually weakly or even negatively coupled.

We found stronger temporal correlations for low-frequency oscillations in Tau+ animals than in Tau- (*F*_(2,59)_ = 27.827; 5 months, p = 0.002; 8 months, p = 1 × 10^−6^) or WT (5 months, p = 0.052; 8 months, p = 5 × 10^−8^) (Figure 5B). We also found that low-frequency power and gamma power was anti-correlated in Tau+ animals, at least at 8 months (*F*_(2,59)_ = 7.915; WT x Tau+, p = 0.002; Tau- x Tau+, p = 0.007; Figure 5B). This anti-correlation suggests that larger sync-index in Tau+ animals reflects presence of epochs in which there is both increased low-frequency power and reduced gamma power.

## Discussion

Tau dysfunction is a hallmark of many neurodegenerative diseases, including AD. We measured the effect of tauopathy on visual cortical oscillations in the rTg4510 mouse model, at earlier and more advanced stages of tauopathy. Our measurements from visual cortex allowed us to characterize the impact of tauopathy across both low- and high-frequency rhythms, at different levels of arousal and movement, and in presence and absence of external stimulation. We observed an overall decrease in gamma power at more advanced stages of tauopathy, and a reduction in stimulus-induced gamma oscillations. Earlier stages of tauopathy, however, were mainly characterized by an increase in low-frequency power during resting state. This increase in low-frequency power arose because Tau+ animals, but not control animals, intermittently entered states of high synchronization, in which low-frequency oscillations were strongly correlated with each other.

### Impairments of gamma-band activity in tauopathy

We found that gamma power was reduced at more advanced stages of tauopathy. This overall reduction in gamma was accompanied by a strong reduction in the amplitude of gamma peaks observed during flickering visual stimulus, but weak if any changes in gamma observed during presentation of a gray screen. The different effects of tauopathy on the two gamma oscillations may be explained by the fact that the two gamma rhythms appear to have different sources: gray-screen gamma-bump in V1 is thought to be inherited from visual thalamic inputs (Saleem et al., 2017) while stimulus-induced gamma-bump may be generated by local V1 circuits (Veit et al., 2017). Expression of mutant human Tau is mainly confined to the neocortex and hippocampus in rTg4510 animals (Santacruz et al., 2005; Tsien et al., 1996) and is largely absent from visual thalamus. Cortical atrophy and cell death increases rapidly in rTg4510 animals from an age of 6 months (Ramsden et al., 2005; Santacruz et al., 2005). By 8 months, cortical atrophy is accompanied by changes in cellular structure: pyramidal cells show loss of dendritic spines and dendritic atrophy (Crimins et al., 2011), and there is a loss of axonal boutons (Jackson et al., 2017, 2020). These morphological changes may result in reduced drive to pyramidal-interneuron networks that are important for the stimulus-induced gamma rhythms (Booth et al., 2016a; Chen et al., 2017; Veit et al., 2017). Our results are consistent with previous recordings in dorsal entorhinal cortex, which showed reduced power at gamma frequencies in 7- to 8-month-old Tau+ rTg4510 animals (Booth et al., 2016a). As previous works suggest there are no changes in the number of inhibitory neurons in Tau+ rTg4510 animals (Booth et al., 2016a; Shimojo et al., 2020), the reduced gamma may instead reflect changes in GABAergic synapses, sub-classes of inhibitory neurons, or the balance of excitation and inhibition in cortical networks (Fu et al., 2019; Maestu et al., 2021; Ranasinghe et al., 2022; Shimojo et al., 2020). Our observations further show little effect of tauopathy on gamma at 5 months of age, suggesting that earlier stages of synaptic dysfunction (before the onset of degeneration) have limited effect on the interneuron networks which are hypothesized to be responsible for gamma rhythms.

### Increased low frequency activity in Tau+ animals during rest

We found that tauopathy had most impact on low-frequency oscillations during rest. Low-frequency oscillations are a prominent feature of inactive states including anesthesia, sleep, and quiet wakefulness (Curto et al., 2009; Greenberg et al., 2008; Poulet and Petersen, 2008; Steriade et al., 1993). These low-frequency states appear to emerge spontaneously in cortical circuits when there is weak drive to the circuit (Harris and Thiele, 2011; Sanchez-Vives et al., 2017). The increase in low-frequency power that we see in tauopathic animals may therefore be explained by a reduced drive to cortical circuits during tauopathy (Menkes-Caspi et al., 2015; Spires-Jones and Hyman, 2014). We find that thalamic inputs to visual cortex (as indexed by narrowband gamma oscillations) appear more resilient to tauopathy in these animals. By contrast, cortical activity is generally reduced during tauopathy (Busche et al., 2019), and previous studies suggest that highly synchronized, low-frequency states are more prevalent when cortical inhibition is relatively strong (Chini et al., 2021; Gao et al., 2017). The increased low-frequency power that we see in tauopathic animals may therefore reflect reduced excitation within visual cortical circuits. In addition, tauopathy may impact oscillations in cortical circuits indirectly, by perturbing neuromodulatory systems (McCormick et al., 2020; Sara, 2009). For example, neurons in locus coeruleus, the source of noradrenergic input to cortex, appear to express Camk2a, which is the promoter for mutant Tau in this tauopathy model (Glennon et al., 2019).

We showed that epochs of synchronization in tauopathic animals are characterized by a large co-fluctuation of activity across a wide range of low frequencies. Fluctuations in delta band (2–4 Hz) were strongly correlated with fluctuations at other low frequencies, up to about 30 Hz, which includes theta and beta bands. Interactions between distal neural circuits are thought to be primarily carried by oscillations in the theta to beta range (Chen et al., 2017; Fournier et al., 2020; Limanowski et al., 2020), and these oscillations may be important in visual recognition (Hayden et al., 2021). The simpler structure of low frequency oscillations may therefore be consistent with a reduction in functional coupling (“disconnection”; (Delbeuck et al., 2003)) of visual cortex to other circuits. Alternatively, the increased low-frequency oscillations may reflect local changes that in turn lead to disconnection, by subsuming other rhythms.

We found that epochs of low-frequency synchronization in tauopathic animals were interleaved with epochs in which cortical oscillations appeared normal. Most models of cortical function suppose that it operates around a critical point, such that cortex can be driven into and out of synchronized states by small perturbations (Poil et al., 2012). Large increases in low-frequency activity may therefore emerge with only a small imbalance in the distribution of excitation and inhibition. The physiological impact of arousal and locomotion is an increase in gamma power and a concomitant decrease in low-frequency power (McCormick and Bal, 1997; Vinck et al., 2015). Indeed, we found that movement was sufficient to abolish the differences between Tau+ and control animals. Intermediate states of arousal (large pupil, stationary) also reduced (but did not abolish) some of the differences between tauopathic and control animals. Our observations suggest that locomotion- or arousal-related modulatory inputs to visual cortex may be sufficient to push tauopathic circuits toward, or into, normal activity ranges.

### Relationship to neurodegeneration and aging

We found that the impact of tauopathy on low-frequency oscillations precedes changes in gamma-range activity. Previous observations in amyloid-β models also showed disruptions to both low-frequency and gamma-range activity (Iaccarino et al., 2016; Scott et al., 2012; Verret et al., 2012), but whether disruptions at lower frequencies also emerge earlier than those at gamma frequencies is not known. Unlike Tau dysfunction, amyloidopathy in known to promote hyperactivity of neuronal ensembles followed by subsequent homeostatic inhibition (Palop et al., 2007), and such imbalances in excitation and inhibition may also lead to increased synchronization (Verret et al., 2012). Theta rhythms are affected prior to significant Amyloid-β dysfunction (Goutagny et al., 2013), and it is therefore possible that impairments to low-frequency oscillations also arise before changes in gamma oscillations in preclinical amyloid models of AD. We found tauopathic degeneration has more impact on specific components of gamma activity compared to others, and it would therefore be of interest to know if there is a similar disaggregation of gamma-range oscillations in preclinical models of amyloidopathy.

EEG measurements in human patients with AD show that the power of low-frequency oscillations is increased in dementia (Coben et al., 1983; Huang et al., 2000; Ranasinghe et al., 2022), and that these changes are more prominent during resting states (Babiloni et al., 2009; de Haan et al., 2008). The gamma activity that normally accompanies visual stimulation is also impaired in patients with mild cognitive impairment or AD (Murty et al., 2021). It is striking that the changes that we see in rTg4510 mouse model are so similar to those observed in human patients with dementia and AD. Measurements of visual cortical function in mouse may therefore provide new opportunities to establish the progression of neurodegeneration; they may also help establish the effectiveness of potential treatments. Recent work suggests that modulation of gamma-range activity using sensory stimulation (visual and auditory) may alleviate progression of neurodegenerative disorders (Adaikkan et al., 2019; Chan et al., 2021; Iaccarino et al., 2016). In this context, it would be useful to know which components of gamma, if any, better track human dementia, as this might offer better therapeutic targets.

Some forms of neurodegeneration, including posterior cortical atrophy (Crutch et al., 2012), are known to have relatively early impact on posterior brain areas. Patients with this “visual variant” present visual impairments associated with parieto-occipital brain dysfunction (Kaeser et al., 2015). In addition, large-scale measurements show Tau accumulation in posterior brain areas of many individuals (Vogel et al., 2021). Measurements of stimulus-induced and spontaneous brain rhythms from visual cortex may therefore be particularly relevant for detecting and tracking tauopathy and degeneration in such individuals. We also observed that increased arousal levels reduced the differences in spectral power between tauopathic and control animals. Better understanding of the relationship between arousal and cortical function may therefore offer new opportunities for understanding neurodegenerative disorders.

### Limitations of the study

We show changes in cortical rhythms in the rTg4510 model of tauopathy. We do not know whether these changes, which we observed in primary visual cortex, extend to other cortical networks or other pre-clinical animal models of tauopathy. We further show that changes in low-frequency oscillations precede changes in gamma-band rhythms in the rTg4510 model. Whether this progressive change in cortical rhythms is recapitulated in other models of tauopathy or in humans requires further investigation. Whether continued mutant Tau expression after 5 months is required for the functional changes we observe at 8 months needs future experiments with appropriately timed interventions.

We confirmed the presence of hyperphosphorylated Tau using AT8 antibody, which detects phosphorylation at the Ser202/Thr205 site and indicates early degenerative changes. However, phosphorylation at this site is reversible, and quantitative comparison of functional to structural changes in rTg4510 would require confirmation of irreversible hyperphosphorylation using other markers, such as AT100 (Ahmed et al., 2020; Kins et al., 2003).

We note that insertion of the P301L transgene in the rTg4510 mouse genome disrupted the *Fgf14* gene, with potential influence on pathology and behavioral abnormalities in this model (Gamache et al., 2019). However, we found no difference in low-frequency or gamma-band rhythms between WT and Tau- animals, suggesting that any off-site effects of the genetic manipulation do not have an impact on our measurements.

## STAR★Methods

### Key resources table

**Table:** 

REAGENT or RESOURCE	SOURCE	IDENTIFIER
**Antibodies**		

Phospho-Tau (Ser202, Thr205) Monoclonal Antibody (AT8)	Thermo Fisher Scientific	Cat# MN1020; RRID: AB_223647

**Chemicals, peptides, and recombinant proteins**		

Phosphate-Buffered saline	Thermo Fisher Scientific	18912014
3,3’-diaminobenzidine	Vector Laboratories	SK-4105
Shandon ClearVue Mountant XYL	Thermo Fisher Scientific	4212
Triton X-100	Sigma	9036-19-5
Sucrose	Sigma	57-50-1
Histoclear	National Diagnostics	A2-0101

**Critical commercial assays**		

Mouse on Mouse Detection Kit	Vector Laboratories	BMK-2202

**Experimental models: Organisms/strains**		

Double Transgenic rTg4510	Envigo	RRID:IMSR_JAX:024854

**Software and algorithms**		

Matlab, R2019a	MathWorks	N/A
OpenEphys	OpenEphys	N/A
SPSS, Version 25	IBM	N/A

### Resource availability

#### Lead contact

Further information and requests for resources and reagents should be directed to and will be fulfilled by the lead contact, Fabio R. Rodrigues (fabio.rodrigues@ucl.ac.uk).

#### Materials availability

This study did not generate new unique reagents.

### Experimental model and subject details

All animals were maintained and experiments were conducted in rooms whose temperature, humidity and noise levels were within the legal limits set out by the Animals (Scientific Procedures) Act 1986 (United Kingdom) and Home Office (United Kingdom) under relevant project and personal licenses. Specifically, the temperature ranged between 19-23°C, the humidity levels ranged between 55% ± 10%, and the noise levels were minimised to remain below 85 dB.

In total, 50 double transgenic rTg4510 (RRID:IMSR_JAX:024854) animals (Ramsden et al., 2005; Santacruz et al., 2005), and 16 wildtype (WT) littermate male mice were obtained at approximately 7 weeks of age from Eli Lilly and Company via Envigo. In order to suppress mutant *tau* expression, 25 transgenic mice received doxycycline treatments from 8 weeks of age, consisting of four 10 mg/kg bolus oral doses of doxycycline (Sigma) in 5% glucose solution by oral gavage across 4 days, followed by *ad libitum* access to Teklad base diet containing 200 ppm doxycycline (Envigo) for the duration of the experiment. These animals were designated as ‘Tau-’, and the remaining 25 transgenic animals as ‘Tau+’. WT and Tau + animals received 4 oral doses of the 5% glucose vehicle solution, followed by *ad libitum* access to standard feed until the end of the experiment. All animals had *ad libitum* access to water. Data were obtained across four interleaved cohorts: 2 cohorts of ‘5 months’ (22–26 weeks, 5 and 7 Tau-, 5 and 6 Tau+, 2 and 4 WT); and 2 cohorts of ‘8 months’ (31–35 weeks, 6 and 7 Tau-, 6 and 7 Tau+, 4 and 6 WT). All animals were group housed at a maximum of 5 animals per cage until 3 days before surgery, and then remained singly housed for the remainder of the experiment. Animals were housed under an inverted 12-h light cycle, and all measurements were carried out during the dark phase of the cycle.

### Method details

#### Surgery

Animals were anaesthetised with 3% isoflurane in a constant flow of oxygen. Preoperative analgesia (Carprieve, 5 mg/kg, Norbrook) was administered subcutaneously, and the eyes protected with lubricant ophthalmic ointment (Xailin, Nicox). Anaesthesia was maintained with 1–2% isoflurane in oxygen, and the depth of the anaesthesia was monitored by paw-pinch-withdrawal reflex and breathing rate. Body temperature was maintained using a heating pad. Animals were placed in a stereotaxic frame (Kopf, Model 940) using non-penetrating ear bars, a craniotomy (<1 mm^2^) was made over the right V1, and a chronic LFP recording electrode (Bear lab chronic microelectrode Monopolar 30070, FHC) was implanted. The coordinates used for 5m WT, Tau-, Tau+ and 8m WT and Tau- mice were 2.8 mm lateral, 0.5 mm anterior from lambda, and 0.45 mm from the cortical surface. For 8 m Tau + mice we used coordinates 2.7 mm lateral, 0.55 mm anterior from lambda, and 0.4 mm from the cortical surface to account for the reduction in brain size in animals with advanced tauopathy. A ground screw was implanted over the left prefrontal cortex. A custom-built stainless-steel metal plate (for subsequent head-fixation) was fixed onto the implant cap using dental cement (Super-Bond C&B, Sun Medical), which was also used to cover the skull, ground screw and metal plate, enclosing and stabilizing the electrode, and fixing the electrode pin on the cap. Postoperative analgesic (Metacam, Boehringer Ingelheim, 1 mg/kg) was mixed in condensed milk for the animals to consume orally for 3 days after the surgery. Animals were allowed to recover for 7 days before testing started.

#### Experimental design

The visual stimulus was a large (80° diameter), sinusoidal grating generated using BonVision (Lopes et al., 2021), and presented on a gamma-corrected computer monitor (Iiyama ProLite EE1890SD). The grating (spatial frequency 0.05 cycles/degree), was oriented either −45° or 45° from vertical (counterbalanced within groups), and was presented in a circular aperture with hard edges, outside of which the monitor was held at the mean luminance (‘grey screen’). The grating was modulated in time by a square-wave waveform and reversed contrast (flickered) at a temporal frequency of 1.0 Hz (i.e. two contrast reversal per second). The display was placed 15 cm from (and normal to) the mouse, and centred on the left monocular visual field. The stimulus was warped to maintain visual angle across the monitor.

All habituation and measurements took place in an experimental room in the same facility as their housing, were transferred this short distance in a covered box, and were returned to the home cage in the housing room at the conclusion of each session. Mice were first habituated to head-fixation in the experimental apparatus over a period of 5 days. During habituation animals were secured by the stainless-steel plate above a styrofoam wheel for increasing periods of time each day (5 min on the first day, 10 min on the second, 20 min on the third, and 30 min on the last two days), and were allowed to walk or run while viewing a grey screen. Movement speed was recorded using a rotary encoder. Pupil size and position were captured via an infrared camera (DMK 22BUC03, ImagingSource; 30 Hz) focused on the left eye through a zoom lens (Computar MLH-10X Macro Zoom Lens), and measured from these videos using custom routines in Bonsai (Lopes et al., 2015). Following habituation, animals were tested daily over 9 consecutive days as described in Papanikolaou et al. (2022). For the purpose of the current study, we analysed recordings made from day 2 to day 8 (7 days total), because the experimental conditions were identical on these days. Each recording session began with a presentation of a grey screen for 3-5 min, followed by the presentation of 10 blocks of the flickering grating, each separated by 30s of grey screen. Each block consisted of 200 reversals, except in 6 of the 5m old animals (2 WT, 2 Tau- and 2 Tau+) where 400 reversals were presented in each block.

LFP signals were acquired, digitised and filtered using an OpenEphys acquisition board (version 1, OpenEphys) and the ground screw provided the reference. The LFP and rotary wheel signals were sampled at 30 kHz, and were synchronised with pupil measurements and visual stimulus via the signal of a photodiode (PDA25K2, Thorlabs, Inc., USA) that monitored timing pulses on a small corner of the monitor shielded from the animal. Portions of the same recordings were used to measure visually-evoked potentials reported elsewhere (Papanikolaou et al., 2022). All data have been reanalysed for the current purposes.

#### Data analysis

All data were analysed using custom software written in MATLAB (version R2019a, MathWorks). Wheel speed was estimated using a quadrature encoder, smoothed with a Gaussian filter (s.d. 50 ms). Eye blinks were identified as instances where eye position exceeded the mean plus twice the variance over the recording session, and the corresponding estimates of pupil size were replaced using nearest-neighbour interpolation. Wheel speed and pupil diameter signals were then mapped to the temporal resolution of spectrograms (described below), and used to define different behavioural epochs for subsequent analysis. Animals were considered stationary when wheel speed was below <1 cm/s. Animals were considered to have a ‘small pupil’ when pupil diameter was smaller than the mean pupil diameter over the recording session.

LFP signals were filtered with an 8th order Chebyshev Type I low-pass filter and then down-sampled to 1 kHz, using the MATLAB function *decimate*. These signals were analysed using the Chronux toolbox for MATLAB (Bokil et al., 2010) using the following parameters: tapers, 3,5; window size, 3s; window shift, 1s. Power spectra were obtained by averaging the resultant spectrograms over relevant epochs. Specific oscillations within the high gamma range were characterized with the ‘fitting oscillations & one over f’ (FOOOF) MATLAB toolbox (Donoghue et al., 2020). Normalized spectral power were produced by subtracting (in dB) FOOOF’s estimate of the aperiodic component from the power spectrum. The parameters used for aperiodic component estimation and peak detection were as follows: peak width limits, [1,8]; max number of peaks to be detected, 8; minimum peak height, 0; threshold for peak detection in standard deviations, 3; aperiodic fitting mode, ‘knee'; frequency range for fitting (in Hz), [2, 100]. The normalized power of specific gamma rhythms was then measured by calculating the mean of the normalized spectra in a 6 Hz window centred on the peak frequency of that rhythm.

To establish the relationship of activity in different frequency bands we calculated the correlation in amplitude over time (amplitude-amplitude coupling). For moving, resting and intermediate states, we extracted relevant epochs from the spectrograms defined above (bin widths: 1s, 0.244 Hz). We calculated Pearson’s correlation coefficient (*r*) for each pair of frequencies, in each session, and then averaged across sessions within animal.

#### Histology

Within 3 days following the last recording session, mice were anaesthetised with isoflurane, and subsequently euthanized with an intraperitoneal overdose of pentobarbital and perfused with 1X phosphate-buffered saline (PBS; Thermo Fisher Scientific). Brains were removed, and weighed following procedure from a previous study (Blackmore et al., 2017) to confirm suppression of the transgene. The right hemisphere was fixed in 10% buffered formalin (7–13 months) for immunohistochemistry. Brains were sunk in PBS +30% sucrose, and 40 μm parasagittal sections were cut on a cryostat (Leica CM1520). Sections were heated in citrate buffer (pH 6.0; Vector Labs) in an oven at 60°C overnight for antigen retrieval. Endogenous peroxidase activity was eliminated by immersing tissue in 0.3% hydrogen peroxide solution (3% in distilled water) for 10 min, followed by washing with PBS with 0.5% Triton X-100 (Sigma). Sections were then incubated with an antibody against phosphorylated Tau (mouse monoclonal AT8, 1:1000; Thermo Fisher Scientific). The Mouse on Mouse Detection Kit (BMK-2202, Vector Laboratories) was used for subsequent steps as described in the supplied protocol. Tissue was then treated for 5 min with 3,3′-diaminobenzidine (DAB; SK-4105, Vector Laboratories). The slides were coverslipped using Shandon ClearVue Mountant XYL (Thermo Fisher Scientific), and digital images obtained using a Leica Microscope (DMi8 S) and camera (DFC7000 GT). Greyscale images were processed (blind to genotype and doxycycline) in MATLAB. In each animal, the mean and variance of pixel intensity was estimated across three regions of interest (ROI) of constant area, each on different sections, that encompassed all layers of primary visual cortex. Visual cortical atrophy was estimated from histological photomicrographs by measuring the orthogonal distance from the pial surface to the end of layer VI of 9 different locations (3 regions from 3 brain slices) within V1 of each animal using Fiji (Schindelin et al., 2012). The depth of the recording electrode was assessed by imaging the electrode track in histological sections. One 5m old Tau + animal was removed from the study because histology showed that the recording electrode was placed too deep.

### Quantification and statistical analysis

All data are presented as mean ± standard error of the mean (s.e.m.). All comparisons were performed on SPSS (version 25, IBM). A two-way or a mixed ANOVA design was applied for all comparisons, using phenotype (Tau-, Tau+, WT) and age (5m, 8m) as between-subjects factors. In cases where a mixed design ANOVA was used, behavioural variables (e.g. locomotion) were used as within-subjects factors, and Greenhouse-Geisser corrections for sphericity were applied. All subsequent post-hoc tests were adjusted for multiple comparisons using the Bonferroni correction. Exact *F* statistics with respective degrees of freedom and *p* values for post-hoc comparisons are presented in the figure legends. All quantifications, descriptions of statistical tests with respective *F* statistics and *p* values for all post-hoc comparisons are described in figure legends and Table S1.

## Data Availability

All data reported in this paper will be shared by the lead contact upon request. This paper does not report original code. Any additional information required to reanalyze the data reported in this paper is available from the lead contact upon request.

## Supplemental information

Document S1. Figure S1

Table S1. Means, standard errors, and statistics for figure data, related to STAR Methods

## References

[bib1] Adaikkan C., Middleton S.J., Marco A., Pao P.C., Mathys H., Kim D.N.W., Gao F., Young J.Z., Suk H.J., Boyden E.S. (2019). Gamma entrainment binds higher-order brain regions and offers neuroprotection. Neuron.

[bib2] Ahmed T., Van der Jeugd A., Caillierez R., Buée L., Blum D., D'Hooge R., Balschun D. (2020). Chronic sodium selenate treatment restores deficits in cognition and synaptic plasticity in a murine model of tauopathy. Front. Mol. Neurosci..

[bib3] Ahnaou A., Moechars D., Raeymaekers L., Biermans R., Manyakov N.V., Bottelbergs A., Wintmolders C., Van Kolen K., Van De Casteele T., Kemp J.A., Drinkenburg W.H. (2017). Emergence of early alterations in network oscillations and functional connectivity in a tau seeding mouse model of Alzheimer's disease pathology. Sci. Rep..

[bib4] Babiloni C., Blinowska K., Bonanni L., Cichocki A., De Haan W., Del Percio C., Dubois B., Escudero J., Fernández A., Frisoni G. (2020). What electrophysiology tells us about Alzheimer's disease: a window into the synchronization and connectivity of brain neurons. Neurobiol. Aging.

[bib5] Babiloni C., Frisoni G., Vecchio F., Lizio R., Pievani M., Geroldi C., Fracassi C., Vernieri F., Ursini F., Rodriguez G. (2009). Global functional coupling of resting EEG rhythms is abnormal in mild cognitive impairment and Alzheimer’s disease. J. Psychophysiol..

[bib82] Bastos, Vezoli, Bosman, Schoffelen, Oostenveld, Dowdall, De Weerd, Kennedy, Fries (2015). Visual areas exert feedforward and feedback influences through distinct frequency channels. Neuron.

[bib6] Blackmore T., Meftah S., Murray T.K., Craig P.J., Blockeel A., Phillips K., Eastwood B., O'Neill M.J., Marston H., Ahmed Z. (2017). Tracking progressive pathological and functional decline in the rTg4510 mouse model of tauopathy. Alzheimers Res. Ther..

[bib7] Bokil H., Andrews P., Kulkarni J.E., Mehta S., Mitra P.P. (2010). Chronux: a platform for analyzing neural signals. J. Neurosci. Methods.

[bib8] Booth C.A., Ridler T., Murray T.K., Ward M.A., de Groot E., Goodfellow M., Phillips K.G., Randall A.D., Brown J.T. (2016). Electrical and network neuronal properties are preferentially disrupted in dorsal, but not ventral, medial entorhinal cortex in a mouse model of tauopathy. J. Neurosci..

[bib9] Booth C.A., Witton J., Nowacki J., Tsaneva-Atanasova K., Jones M.W., Randall A.D., Brown J.T. (2016). Altered intrinsic pyramidal neuron properties and pathway-specific synaptic dysfunction underlie aberrant hippocampal network function in a mouse model of tauopathy. J. Neurosci..

[bib10] Bruns A., Eckhorn R., Jokeit H., Ebner A. (2000). Amplitude envelope correlation detects coupling among incoherent brain signals. Neuroreport.

[bib11] Busche M.A., Wegmann S., Dujardin S., Commins C., Schiantarelli J., Klickstein N., Kamath T.V., Carlson G.A., Nelken I., Hyman B.T. (2019). Tau impairs neural circuits, dominating amyloid-beta effects, in Alzheimer models in vivo. Nat. Neurosci..

[bib12] Buzsáki G., Draguhn A. (2004). Neuronal oscillations in cortical networks. Science.

[bib13] Chan D., Suk H.-J., Jackson B., Milman N.P., Stark D., Klerman E.B., Kitchener E., Avalos V.S.F., Banerjee A., Beach S.D. (2021). 40Hz sensory stimulation induces gamma entrainment and affects brain structure, sleep and cognition in patients with Alzheimer’s dementia. medRxiv.

[bib14] Chen G., Zhang Y., Li X., Zhao X., Ye Q., Lin Y., Tao H.W., Rasch M.J., Zhang X. (2017). Distinct inhibitory circuits orchestrate cortical beta and gamma band oscillations. Neuron.

[bib15] Chini M., Pfeffer T., Hanganu-Opatz I.L. (2021). Developmental increase of inhibition drives decorrelation of neural activity. bioRxiv.

[bib16] Ciupek S.M., Cheng J., Ali Y.O., Lu H.C., Ji D. (2015). Progressive functional impairments of hippocampal neurons in a tauopathy mouse model. J. Neurosci..

[bib17] Coben L.A., Danziger W.L., Berg L. (1983). Frequency analysis of the resting awake EEG in mild senile dementia of Alzheimer type. Electroencephalogr. Clin. Neurophysiol..

[bib18] Crimins J.L., Rocher A.B., Peters A., Shultz P., Lewis J., Luebke J.I. (2011). Homeostatic responses by surviving cortical pyramidal cells in neurodegenerative tauopathy. Acta Neuropathol..

[bib19] Crutch S.J., Lehmann M., Schott J.M., Rabinovici G.D., Rossor M.N., Fox N.C. (2012). Posterior cortical atrophy. Lancet Neurol..

[bib20] Curto C., Sakata S., Marguet S., Itskov V., Harris K.D. (2009). A simple model of cortical dynamics explains variability and state dependence of sensory responses in urethane-anesthetized auditory cortex. J. Neurosci..

[bib21] Das M., Maeda S., Hu B., Yu G.Q., Guo W., Lopez I., Yu X., Tai C., Wang X., Mucke L. (2018). Neuronal levels and sequence of tau modulate the power of brain rhythms. Neurobiol. Dis..

[bib22] de Haan W., Stam C.J., Jones B.F., Zuiderwijk I.M., van Dijk B.W., Scheltens P. (2008). Resting-state oscillatory brain dynamics in Alzheimer disease. J. Clin. Neurophysiol..

[bib23] Delbeuck X., Van der Linden M., Collette F. (2003). Alzheimer's disease as a disconnection syndrome?. Neuropsychol. Rev..

[bib24] Donoghue T., Haller M., Peterson E.J., Varma P., Sebastian P., Gao R., Noto T., Lara A.H., Wallis J.D., Knight R.T. (2020). Parameterizing neural power spectra into periodic and aperiodic components. Nat. Neurosci..

[bib25] Engel A.K., Fries P., Singer W. (2001). Dynamic predictions: oscillations and synchrony in top-down processing. Nat. Rev. Neurosci..

[bib26] Fournier J., Saleem A.B., Diamanti E.M., Wells M.J., Harris K.D., Carandini M. (2020). Mouse visual cortex is modulated by distance traveled and by theta oscillations. Curr. Biol..

[bib27] Fu H., Possenti A., Freer R., Nakano Y., Hernandez Villegas N.C., Tang M., Cauhy P.V.M., Lassus B.A., Chen S., Fowler S.L. (2019). A tau homeostasis signature is linked with the cellular and regional vulnerability of excitatory neurons to tau pathology. Nat. Neurosci..

[bib28] Gamache J., Benzow K., Forster C., Kemper L., Hlynialuk C., Furrow E., Ashe K.H., Koob M.D. (2019). Factors other than hTau overexpression that contribute to tauopathy-like phenotype in rTg4510 mice. Nat. Commun..

[bib29] Gao R., Peterson E.J., Voytek B. (2017). Inferring synaptic excitation/inhibition balance from field potentials. Neuroimage.

[bib30] Glennon E., Carcea I., Martins A.R.O., Multani J., Shehu I., Svirsky M.A., Froemke R.C. (2019). Locus coeruleus activation accelerates perceptual learning. Brain Res..

[bib31] Goutagny R., Gu N., Cavanagh C., Jackson J., Chabot J.G., Quirion R., Krantic S., Williams S. (2013). Alterations in hippocampal network oscillations and theta-gamma coupling arise before Abeta overproduction in a mouse model of Alzheimer's disease. Eur. J. Neurosci..

[bib32] Green C., Sydow A., Vogel S., Anglada-Huguet M., Wiedermann D., Mandelkow E., Mandelkow E.M., Hoehn M. (2019). Functional networks are impaired by elevated tau-protein but reversible in a regulatable Alzheimer's disease mouse model. Mol. Neurodegener..

[bib33] Greenberg D.S., Houweling A.R., Kerr J.N.D. (2008). Population imaging of ongoing neuronal activity in the visual cortex of awake rats. Nat. Neurosci..

[bib34] Harris K.D., Thiele A. (2011). Cortical state and attention. Nat. Rev. Neurosci..

[bib35] Hayden D.J., Montgomery D.P., Cooke S.F., Bear M.F. (2021). Visual recognition is heralded by shifts in local field potential oscillations and inhibitory networks in primary visual cortex. J. Neurosci..

[bib36] Holton C.M., Hanley N., Shanks E., Oxley P., McCarthy A., Eastwood B.J., Murray T.K., Nickerson A., Wafford K.A. (2020). Longitudinal changes in EEG power, sleep cycles and behaviour in a tau model of neurodegeneration. Alzheimers Res. Ther..

[bib37] Hoover B.R., Reed M.N., Su J., Penrod R.D., Kotilinek L.A., Grant M.K., Pitstick R., Carlson G.A., Lanier L.M., Yuan L.L. (2010). Tau mislocalization to dendritic spines mediates synaptic dysfunction independently of neurodegeneration. Neuron.

[bib38] Huang C., Wahlund L., Dierks T., Julin P., Winblad B., Jelic V. (2000). Discrimination of Alzheimer's disease and mild cognitive impairment by equivalent EEG sources: a cross-sectional and longitudinal study. Clin. Neurophysiol..

[bib39] Iaccarino H.F., Singer A.C., Martorell A.J., Rudenko A., Gao F., Gillingham T.Z., Mathys H., Seo J., Kritskiy O., Abdurrob F. (2016). Gamma frequency entrainment attenuates amyloid load and modifies microglia. Nature.

[bib40] Jackson J.S., Johnson J.D., Meftah S., Murray T.K., Ahmed Z., Fasiolo M., Hutton M.L., Isaac J.T.R., O’Neill M.J., Ashby M.C. (2020). Differential aberrant structural synaptic plasticity in axons and dendrites ahead of their degeneration in tauopathy. bioRxiv.

[bib41] Jackson J.S., Witton J., Johnson J.D., Ahmed Z., Ward M., Randall A.D., Hutton M.L., Isaac J.T., O'Neill M.J., Ashby M.C. (2017). Altered synapse stability in the early stages of tauopathy. Cell Rep..

[bib42] Kaeser P.F., Ghika J., Borruat F.X. (2015). Visual signs and symptoms in patients with the visual variant of Alzheimer disease. BMC Ophthalmol..

[bib43] Kins S., Kurosinski P., Nitsch R.M., Götz J. (2003). Activation of the ERK and JNK signaling pathways caused by neuron-specific inhibition of PP2A in transgenic mice. Am. J. Pathol..

[bib44] Kopeikina K.J., Polydoro M., Tai H.C., Yaeger E., Carlson G.A., Pitstick R., Hyman B.T., Spires-Jones T.L. (2013). Synaptic alterations in the rTg4510 mouse model of tauopathy. J. Comp. Neurol..

[bib45] Limanowski J., Litvak V., Friston K. (2020). Cortical beta oscillations reflect the contextual gating of visual action feedback. Neuroimage.

[bib46] Lopes G., Bonacchi N., Frazão J., Neto J.P., Atallah B.V., Soares S., Moreira L., Matias S., Itskov P.M., Correia P.A. (2015). Bonsai: an event-based framework for processing and controlling data streams. Front. Neuroinform..

[bib47] Lopes G., Farrell K., Horrocks E.A., Lee C.Y., Morimoto M.M., Muzzu T., Papanikolaou A., Rodrigues F.R., Wheatcroft T., Zucca S. (2021). Creating and controlling visual environments using BonVision. Elife.

[bib48] Maestú F., de Haan W., Busche M.A., DeFelipe J. (2021). Neuronal excitation/inhibition imbalance: core element of a translational perspective on Alzheimer pathophysiology. Ageing Res. Rev..

[bib49] McAfee S.S., Liu Y., Dhamala M., Heck D.H. (2018). Thalamocortical communication in the awake mouse visual system involves phase synchronization and rhythmic spike synchrony at high gamma frequencies. Front. Neurosci..

[bib50] McCormick D.A., Bal T. (1997). Sleep and arousal: thalamocortical mechanisms. Annu. Rev. Neurosci..

[bib51] McCormick D.A., Nestvogel D.B., He B.J. (2020). Neuromodulation of brain state and behavior. Annu. Rev. Neurosci..

[bib52] Menkes-Caspi N., Yamin H.G., Kellner V., Spires-Jones T.L., Cohen D., Stern E.A. (2015). Pathological tau disrupts ongoing network activity. Neuron.

[bib53] Murty D.V., Manikandan K., Kumar W.S., Ramesh R.G., Purokayastha S., Nagendra B., Ml A., Balakrishnan A., Javali M., Rao N.P., Ray S. (2021). Stimulus-induced gamma rhythms are weaker in human elderly with mild cognitive impairment and Alzheimer's disease. Elife.

[bib54] Niell C.M., Stryker M.P. (2010). Modulation of visual responses by behavioral state in mouse visual cortex. Neuron.

[bib55] Palop J.J., Chin J., Roberson E.D., Wang J., Thwin M.T., Bien-Ly N., Yoo J., Ho K.O., Yu G.Q., Kreitzer A. (2007). Aberrant excitatory neuronal activity and compensatory remodeling of inhibitory hippocampal circuits in mouse models of Alzheimer's disease. Neuron.

[bib56] Papanikolaou A., Rodrigues F.R., Holeniewska J., Phillips K.G., Saleem A.B., Solomon S.G. (2022). Plasticity in visual cortex is disrupted in a mouse model of tauopathy. Commun. Biol..

[bib57] Poil S.S., Hardstone R., Mansvelder H.D., Linkenkaer-Hansen K. (2012). Critical-state dynamics of avalanches and oscillations jointly emerge from balanced excitation/inhibition in neuronal networks. J. Neurosci..

[bib58] Poulet J.F.A., Petersen C.C.H. (2008). Internal brain state regulates membrane potential synchrony in barrel cortex of behaving mice. Nature.

[bib59] Ramsden M., Kotilinek L., Forster C., Paulson J., McGowan E., SantaCruz K., Guimaraes A., Yue M., Lewis J., Carlson G. (2005). Age-dependent neurofibrillary tangle formation, neuron loss, and memory impairment in a mouse model of human tauopathy (P301L). J. Neurosci..

[bib60] Ranasinghe K.G., Verma P., Cai C., Xie X., Kudo K., Gao X., Lerner H., Mizuiri D., Strom A., Iaccarino L. (2022). Altered excitatory and inhibitory neuronal subpopulation parameters are distinctly associated with tau and amyloid in Alzheimer's disease. Elife.

[bib61] Reimer J., Froudarakis E., Cadwell C.R., Yatsenko D., Denfield G.H., Tolias A.S. (2014). Pupil fluctuations track fast switching of cortical states during quiet wakefulness. Neuron.

[bib62] Roy S., Zhang B., Lee V.M.Y., Trojanowski J.Q. (2005). Axonal transport defects: a common theme in neurodegenerative diseases. Acta Neuropathol..

[bib63] Saleem A.B., Lien A.D., Krumin M., Haider B., Rosón M.R., Ayaz A., Reinhold K., Busse L., Carandini M., Harris K.D. (2017). Subcortical source and modulation of the narrowband gamma oscillation in mouse visual cortex. Neuron.

[bib64] Sanchez-Vives M.V., Massimini M., Mattia M. (2017). Shaping the default activity pattern of the cortical network. Neuron.

[bib65] Santacruz K., Lewis J., Spires T., Paulson J., Kotilinek L., Ingelsson M., Guimaraes A., DeTure M., Ramsden M., McGowan E. (2005). Tau suppression in a neurodegenerative mouse model improves memory function. Science.

[bib66] Sara S.J. (2009). The locus coeruleus and noradrenergic modulation of cognition. Nat. Rev. Neurosci..

[bib67] Schindelin J., Arganda-Carreras I., Frise E., Kaynig V., Longair M., Pietzsch T., Preibisch S., Rueden C., Saalfeld S., Schmid B. (2012). Fiji: an open-source platform for biological-image analysis. Nat. Methods.

[bib68] Scott L., Feng J., Kiss T., Needle E., Atchison K., Kawabe T.T., Milici A.J., Hajós-Korcsok E., Riddell D., Hajós M. (2012). Age-dependent disruption in hippocampal theta oscillation in amyloid-beta overproducing transgenic mice. Neurobiol. Aging.

[bib69] Senzai Y., Fernandez-Ruiz A., Buzsáki G. (2019). Layer-specific physiological features and interlaminar interactions in the primary visual cortex of the mouse. Neuron.

[bib70] Shimojo M., Takuwa H., Takado Y., Tokunaga M., Tsukamoto S., Minatohara K., Ono M., Seki C., Maeda J., Urushihata T. (2020). Selective disruption of inhibitory synapses leading to neuronal hyperexcitability at an early stage of tau pathogenesis in a mouse model. J. Neurosci..

[bib71] Siegel M., Donner T.H., Engel A.K. (2012). Spectral fingerprints of large-scale neuronal interactions. Nat. Rev. Neurosci..

[bib72] Spires-Jones T.L., Hyman B.T. (2014). The intersection of amyloid beta and tau at synapses in Alzheimer's disease. Neuron.

[bib73] Steriade M., McCormick D.A., Sejnowski T.J. (1993). Thalamocortical oscillations in the sleeping and aroused brain. Science.

[bib74] Storchi R., Bedford R.A., Martial F.P., Allen A.E., Wynne J., Montemurro M.A., Petersen R.S., Lucas R.J. (2017). Modulation of fast narrowband oscillations in the mouse retina and dLGN according to background light intensity. Neuron.

[bib75] Timofeev I., Schoch S.F., LeBourgeois M.K., Huber R., Riedner B.A., Kurth S. (2020). Spatio-temporal properties of sleep slow waves and implications for development. Curr. Opin. Physiol..

[bib76] Tsien J.Z., Chen D.F., Gerber D., Tom C., Mercer E.H., Anderson D.J., Mayford M., Kandel E.R., Tonegawa S. (1996). Subregion- and cell type-restricted gene knockout in mouse brain. Cell.

[bib77] Veit J., Hakim R., Jadi M.P., Sejnowski T.J., Adesnik H. (2017). Cortical gamma band synchronization through somatostatin interneurons. Nat. Neurosci..

[bib78] Verret L., Mann E.O., Hang G.B., Barth A.M.I., Cobos I., Ho K., Devidze N., Masliah E., Kreitzer A.C., Mody I. (2012). Inhibitory interneuron deficit links altered network activity and cognitive dysfunction in Alzheimer model. Cell.

[bib79] Vinck M., Batista-Brito R., Knoblich U., Cardin J.A. (2015). Arousal and locomotion make distinct contributions to cortical activity patterns and visual encoding. Neuron.

[bib80] Vogel J.W., Young A.L., Oxtoby N.P., Smith R., Ossenkoppele R., Strandberg O.T., La Joie R., Aksman L.M., Grothe M.J., Iturria-Medina Y. (2021). Four distinct trajectories of tau deposition identified in Alzheimer's disease. Nat. Med..

[bib81] Vyazovskiy V.V., Olcese U., Hanlon E.C., Nir Y., Cirelli C., Tononi G. (2011). Local sleep in awake rats. Nature.

