# Supplementary material for: Altered low-frequency brain rhythms precede changes in gamma power during tauopathy
Source: iScience. 2022 Sep 28;25(10):105232. doi: 10.1016/j.isci.2022.105232 (PMC9579020; doi:10.1016/j.isci.2022.105232)
Supplement: Document S1. Figure S1 [file mmc1.pdf]

iScience, Volume *25*

Supplemental information

Altered low-frequency brain rhythms precede changes in gamma power during tauopathy

Fabio R. Rodrigues, Amalia Papanikolaou, Joanna Holeniewska, Keith G. Phillips, Aman B. Saleem, and Samuel G. Solomon

Supplementary Information

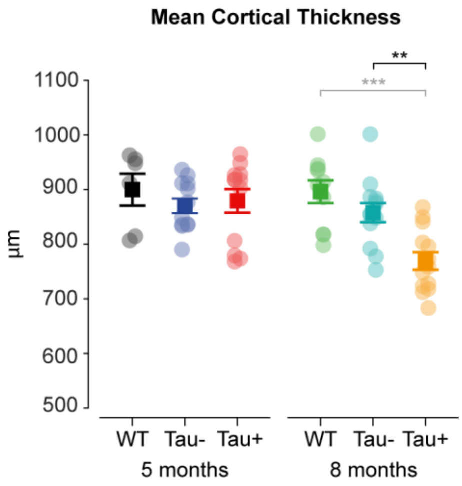

Figure S1. Atrophy in primary visual cortex of tauopathic mice. Related to Figure 1. We estimated the thickness of visual cortex (V1) by measuring the distance between the pia and the bottom of layer VI. Each transparent datapoint shows the average cortical thickness obtained across 9 locations (3 locations in each of 3 slices) in each mouse. Opaque symbols show mean ± 1 s.e.m. across mice in each group. At 5 months, the cortical thickness of Tau+ animals (879.3±21.4 µm; mean±s.e.m) was similar to that of Tau- (870.3±13.4 µm) and WT (899.8±29.1 µm) ($F_{(5,60)}$=6.793, P=1.00). At 8 months, V1 was significantly thinner in Tau+ animals (769.2±16.2 µm) compared to both Tau- (857.8.8±17.5 µm; P=0.002) and WT (896.1±20.9 µm; P=3.3x10^{-5}). All comparisons were performed using a two-way ANOVA. ** P<0.01; *** P<0.001.

Table S1. Means, standard errors, and statistics for figure data. Related to STAR Methods. Excel file containing the means, standard errors, and statistics for data presented and analysed in each individual main figure. Each sheet contains the respective values for one of the figures.
